# Dendritic cell activation and cytokine response in vaccine breakthrough TBE patients after *in vitro* stimulation with TBEV

**DOI:** 10.3389/fimmu.2023.1190803

**Published:** 2023-05-16

**Authors:** Miša Marušić, Andreja Nataša Kopitar, Miša Korva, Nataša Knap, Petra Bogovič, Franc Strle, Alojz Ihan, Tatjana Avšič-Županc

**Affiliations:** ^1^ Laboratory for Diagnostics of Zoonoses and World Health Organisation (WHO) Center, Institute of Microbiology and Immunology, Faculty of Medicine, University of Ljubljana, Ljubljana, Slovenia; ^2^ Laboratory for Cellular Immunology, Institute of Microbiology and Immunology, Faculty of Medicine, University of Ljubljana, Ljubljana, Slovenia; ^3^ Department of Infectious Diseases, University Medical Center Ljubljana, Ljubljana, Slovenia

**Keywords:** tick-borne encephalitis, vaccine breakthrough, cytokines, dendritic cells, immune response *in vitro*

## Abstract

Tick-borne encephalitis (TBE) is a viral infection of the human central nervous system caused by the TBE virus (TBEV). The most effective protective measure against TBE is vaccination. Despite the highly immunogenic vaccine, cases of vaccine breakthroughs (VBTs) occur. One of the first targets of infection is dendritic cells (DC), which represent a fundamental bridge between innate and adaptive immunity through antigen presentation, costimulation, and cytokine production. Therefore, we investigated the activation and maturation of DCs and cytokine production after *in vitro* TBEV stimulation of peripheral blood mononuclear cells (PBMCs) obtained from VBT and unvaccinated TBE patients. Our results showed that the expression of HLA-DR and CD86 on DCs, was upregulated to a similar extent in both vaccinated and unvaccinated TBE patients but differed in cytokine production after stimulation with TBEV. PBMCs from patients with VBT TBE responded with lower levels of IFN-α and the proinflammatory cytokines IL-12 (p70) and IL-15 after 24- and 48-hour *in vitro* stimulation with TBEV, possibly facilitating viral replication and influencing the development of cell-mediated immunity. On the other hand, significantly higher levels of IL-6 in addition to an observed trend of higher expression of TNF-α measured after 6 days of *in vitro* stimulation of PBMC could support disruption of the blood–brain barrier and promote viral and immune cell influx into the CNS, leading to more severe disease in VBT TBE patients.

## 1 Introduction

Tick-borne encephalitis (TBE) is a disease of the central nervous system (CNS) caused by the tick-borne encephalitis virus (TBEV). TBEV is usually transmitted through an infected tick bite, but humans can also be infected by consumption of unpasteurized milk or dairy products from infected livestock (1, 2). The majority of infections with the European subtype of TBEV (Eu-TBEV) are asymptomatic, whereas 56% to 87% of clinically manifested infections follow a biphasic course (3–7).

Currently there is no etiologic therapy for TBE; treatment is mainly supportive. The most effective protection against TBE is regular vaccination, which successfully promotes the formation of neutralizing antibodies in 98% to 99.5% of vaccinated people (8, 9). Nevertheless, 39 TBE vaccine breakthrough (VBT) cases were reported between 2000 and 2015 in Slovenia (10). In patients with VBT TBE, the onset of neurological symptoms is accompanied by high levels of specific and neutralizing IgG, whereas IgM antibodies are absent or present only in low levels (10–12). This indicates an anamnestic immune response, but one that is likely insufficient to prevent disease. Additionally, VBT TBE patients have significantly elevated levels of proinflammatory cytokines upon admission to the hospital, specifically those associated with blood-brain barrier (BBB) impairment. This finding suggests that immune cell infiltration into the central nervous system (CNS) and the development of intrathecal inflammation may be involved in the pathogenesis of VBT TBE (13).

An adequate immune response in the early stages of infection significantly limits viral replication and spread (14, 15). Some of the first cells that come into contact with TBEV after a tick bite are thought to be dendritic cells (DCs) (16), which could thus facilitate viral spread throughout the body. Antigen uptake by DCs and its recognition by pathogen recognition receptors (PRR) triggers DC maturation and activation, leading to increased expression of the surface activation markers HLA-DR and CD86 as well as increased secretion of specific cytokines (17). Because mature DCs are responsible for initiating several antiviral defense mechanisms, including T cell activation and proliferation through antigen presentation, costimulation, and cytokine secretion, as well as isotype switching and B cell differentiation into antibody-secreting plasma cells (18, 19), they represent an important link between the innate and adaptive immune system. As the infection of DCs by several other flaviviruses—including Japanese encephalitis virus (20), dengue virus (DENV) (21), and West Nile virus (WNV) (22)—results in altered DC maturation and T cell proliferation, we hypothesize that viral interaction with DCs may potentially also affect TBE pathogenesis in VBT patients.

To gain a better understanding of the events in TBE pathogenesis, we stimulated PBMCs obtained from vaccinated and unvaccinated TBE patients with TBEV *in vitro* and measured DC activation through expression of activation and maturation markers and proinflammatory cytokine production.

## 2 Materials and methods

### 2.1 Study design and subjects

All TBE patients enrolled in our study were diagnosed between January 2003 and December 2018 at the Department of Infectious Diseases, University Medical Center Ljubljana, Slovenia. Following disease remission, patients were invited for a follow-up examination in which 15 ml of peripheral whole blood was collected for isolation of PBMCs. Of them, 10 VBT TBE patients that had received complete basic vaccination with or without booster doses and without delays in administration and 10 sex- and age-matched unvaccinated patients diagnosed with TBE in the same year were included in our study. Characteristics of TBE patients enrolled in our study are shown in **Table 1**.

**Table 1:** Characteristics of VBT TBE patients and unvaccinated TBE patients enrolled in the study.

Characteristics		VBT TBE	unvaccinated TBE
**Gender (N)**	**F**	4	4
	**M**	6	6
**Age at TBE onset (years)**		62,0 (56,0-64,8)	60,0 (56,3-66,3)
**Complete TBE vaccination (3 doses)**		10 (100%)	NA
**Booster dose (N, %)**		4 (40%)	NA
**Time interval between last TBE vaccine dose and TBE onset (years)**		3,1 (1,8-3,9)	NA
**Age at PBMC collection (years)**		70,5 (64,8-73,0)	69,5 (66,5-73,3)
**Time between TBE onset and PBMC collection (years)**		7,5 (5,3-9,8)	8,5 (6,3-10,8)

Data are shown median (interquartile range, IQR) or number (%). F, female; M, male; NA, not applicable; PBMC, peripheral blood mononuclear cells; TBE, tick borne encephalitis.

PBMCs were isolated from blood samples with ethylenediaminetetraacetic acid (EDTA) using Ficoll-Paque PLUS (GE Healthcare, Uppsala, Sweden) density gradient centrifugation. Briefly, whole blood was mixed with Roswell Park Memorial Institute (RPMI) 1640 Medium in a 1:2 ratio and centrifuged in Leucosep tubes (Greiner Bio-One, GmbH, Germany) containing Ficoll-Paque PLUS at 645 × *g* for 10 minutes at 21 °C without brake. The buffy coat containing the PBMCs was collected, washed twice in 10 ml of fresh RPMI-1640 and resuspended and stored in RPMI-1640 (10%) + FCS (80%) + dimethyl sulfoxide (DMSO; 10%) before being aliquoted and stored at −80 °C until further use.

### 2.2 Virus preparation

TBEV, Ljubljana strain (EVA-GLOBAL-Ref-SKU: 007V-02198), was used as the stock virus. To prepare the virus, the VERO E6 cell line was cultured in complete Dulbecco’s modified Eagle’s medium (DMEM; GIBCO, Invitrogen, Massachusetts, United States), supplemented with 10% fetal bovine serum (FBS). Prior to inoculation, the cell supernatant was removed, and 1 ml of TBEV was transferred onto 24-hour-old VERO E6 cells (Vero 76, clone E6; ATCC-LGC, Virginia, USA), with 80% confluency. The cells were incubated for one hour at 37 °C and 5% CO_2_, followed by the addition of 10 ml of DMEM supplemented with 4% FBS. The cells were further incubated for seven days under the same conditions.

After the incubation period, the supernatant was transferred to a 15 ml tube. The remaining adherent cells were detached using glass beads in 5 ml of filtered saline solution supplemented with 5% FBS. The virus was then centrifuged using Amicon Ultra Centrifugal Filter Units with an ultrafiltration membrane (Merck KgaA, Darmstadt, Germany) as per the manufacturer’s instructions. The virus was transferred to the centrifugal filter unit and centrifuged at 4,000 × g for 30 minutes. The ultrafiltration membrane was then transferred to a new tube, 15 ml of RPMI-1640 medium was added, and the centrifugation procedure was repeated. The virus was resuspended in 30 ml of RPMI-1640 medium and stored in 1 ml aliquots at −80 °C until further use.

### 2.3 Virus quantification

Prepared TBEV stock was quantified using the median tissue culture infectious dose (TCID_50_) assay on VERO E6 cells. Briefly, TBEV was serially diluted (10-fold) in Dulbecco’s Modified Eagle Medium (DMEM) with 10% FBS and 50 μl of each dilution was added to VERO E6 cells in a 96-well cell culture plate, except for the last three columns which represented a negative control. After 7 days of incubation at 37 °C and 5% CO_2_, the cells were fixed with 100 μl of 4% formaldehyde and stained with 1% crystal violet. The TCID_50_ was calculated using the Spearman-Kärber method based on the dilution at which 50% of wells showed cytopathic effects.

### 2.4 *In vitro* stimulation of PBMCs with TBEV

On the day of the experiment, cryotubes containing PBMCs were thawed in a 37 °C water bath. The PBMCs were transferred to 15 ml tubes and warm RPMI-1640 supplemented with 10% inactivated human serum (IHS; Merck KgaA, Darmstadt, Germany) was slowly added with constant stirring. After centrifugation at 287 × *g* for 10 minutes at 21 °C, the cells were washed twice with 10% IHS in RPMI-1640, and then resuspended in 10% IHS in RPMI-1640. Cell viability was determined using trypan blue on the Countess Automated Cell Counter (Thermo Fisher Scientific, Carlsbad, CA, USA).

A working concentration of 2 × 10^6^ viable cells/ml was prepared in RPMI-1640 containing 10% IHS (Merck KgaA, Darmstadt, Germany), 1% L-glutamine (Thermo Fisher Scientific, Carlsbad, CA, USA), 1% penicillin (100 U/ml), and 1% streptomycin (0.1 mg/ml), and 500 μl of cell suspension was seeded into 24-well cell culture plates. PBMCs were stimulated with TBEV with multiplicity of infection (MOI) 2 for 24 hours, 48 hours, and 6 days at 37 °C and 5% CO_2_. Unstimulated PBMCs from each individual at each time point represented the negative control.

At each stimulation time point, PBMCs were harvested and centrifuged at 287 × *g* for 5 min at room temperature to separate the supernatant for cytokine analysis. The supernatant was stored at −80 °C for cytokine analysis, and the pellet was resuspended in 250 μl of cell culture medium. Adherent cells were detached using ice-cold 2 mM EDTA in PBS, centrifuged at 287 × *g* for 5 min at room temperature and combined with the harvested cells from the first tube. The cells were thoroughly mixed, and the samples were immediately prepared for flow cytometric analysis.

### 2.5 Immunostaining and flow cytometry analysis

Before immunostaining, the viability of stimulated and unstimulated PBMCs was measured with trypan blue on the Countess II Automated Cell Counter (Thermo Fisher Scientific, Carlsbad, CA, USA) according to the manufacturer’s instructions. Fluorescently labeled monoclonal antibodies (mAb) to cell surface antigens used for immunostaining of PBMCs included PE-conjugated mouse anti-human CD86 (clone 2331), PerCP Cy5.5-conjugated mouse anti-human CD3 (clone SK7), CD14 (clone M5E2), CD16 (clone 3G8) and CD19 (clone SJ25C1), PE Cy7-conjugated mouse anti-human CD123 (clone 7G3), APC-conjugated mouse anti-human CD11c (clone MJ4-27G12), APC Cy7-conjugated mouse anti-human HLA-DR (clone L243), and BV510-conjugated mouse anti-human CD1c (clone F10/21A3), purchased from BD Biosciences (San Jose, CA, USA), whereas FITC-conjugated mouse anti-human CD303 (clone AC144) and ViolBlue-conjugated mouse anti-human CD141 (clone AD5-14H12) were purchased from Miltenyi Biotec (Bergisch Gladbach, Germany). A mAb combination was added to a 150 μl PBMC suspension collected 24 and 48 hours after TBEV stimulation and incubated for 20 min in the dark. The cells were then fixed using 2 ml of lysis buffer (Becton Dickinson, Franklin Lakes, NJ, USA) for 10 min in the dark. After 5 min centrifugation at 287 × *g*, PBMCs were washed twice with PBS and resuspended in 250 μl of PBS. Samples were acquired on a FACS Canto II flow cytometer (BD Biosciences, San Jose, CA, USA) and the results were analyzed with FACS Diva software (BD Biosciences, San Jose, CA, USA).

A minimum of 100,000 events were recorded with the flow cytometer. Doublets and dead cells were eliminated based on forward/side scatter plots, and DCs were gated based on high HLA-DR expression and the absence of surface expression of lineage markers CD3, CD14, CD16, and CD19. From the population of DCs, subpopulations were further divided based on the expression of specific surface. The combination of CD11c and CD141 was used to determine cDC1, CD11c and CD1c for cDC2, and CD123 and CD303 for pDC. FMO controls were included to define the negative gates for selected populations. The median fluorescence intensity (MFI) of HLA-DR and CD86 on each subpopulation of DCs was analyzed and served as a marker for activation after *in vitro* stimulation with TBEV. The gating strategy and fluorescence minus one (FMO) controls are shown in **Supplementary Figure 1**.

### 2.6 Cytokine quantification with a multiplex immunoassay

Cytokine concentrations in PBMC supernatants were measured using commercially available kits for the detection of soluble immune mediators: Human Cytokine/Chemokine Magnetic Bead Panel and Human Cytokine/Chemokine Magnetic Bead Panel IV (Millipore, Darmstadt, Germany) for the Luminex platform on a Magpix instrument (Luminex, Austin, TX, USA) according to the manufacturer’s instructions. The immunoassays detect IFN-α, IFN-β, IL-1β, IL-6, IL-12 (p70), IL-15, and TNF-α. Briefly, cell culture supernatants were thawed, centrifuged at 1,000 × *g* for 5 min, and diluted 1:3 with Assay Buffer (Millipore, Germany) provided in the multiplex assay kits. All measurements with a single panel were performed on the same day in one complete experiment to minimize inter-assay variation. Data analysis was performed using Milliplex Analyst 5.1 software. All values outside the upper and lower ends of the standard curve were considered maximum and minimum values, respectively.

### 2.7 Statistical analysis

Differences between two independent groups were analyzed using the Mann–Whitney test in GraphPad Prism 7. Each group was first compared to a negative control to determine the presence of a response, and subsequently, the extent of the response was compared between the two groups. Values with *p* ≤ 0.05 were interpreted as statistically significant; statistical significance levels are indicated by the number of asterisks: *p ≤ 0.05, **p ≤ 0.01, ***p ≤ 0.001, and ****p ≤ 0.0001.

## 3 Results

### 3.1 DCs increase expression of HLA-DR and CD86 after TBEV stimulation *in vitro*

Due to nonspecific symptoms in the first phase of the disease, the early immune response to TBEV infection is poorly investigated. To investigate the activation status of DC after encountering TBEV, we stimulated PBMCs from VBT and unvaccinated TBE patients *in vitro* with TBEV (MOI 2) for 24 and 48 hours and measured the expression of the activation and maturation markers HLA-DR and CD86 on specific DC subpopulations.

**Figure 1:**
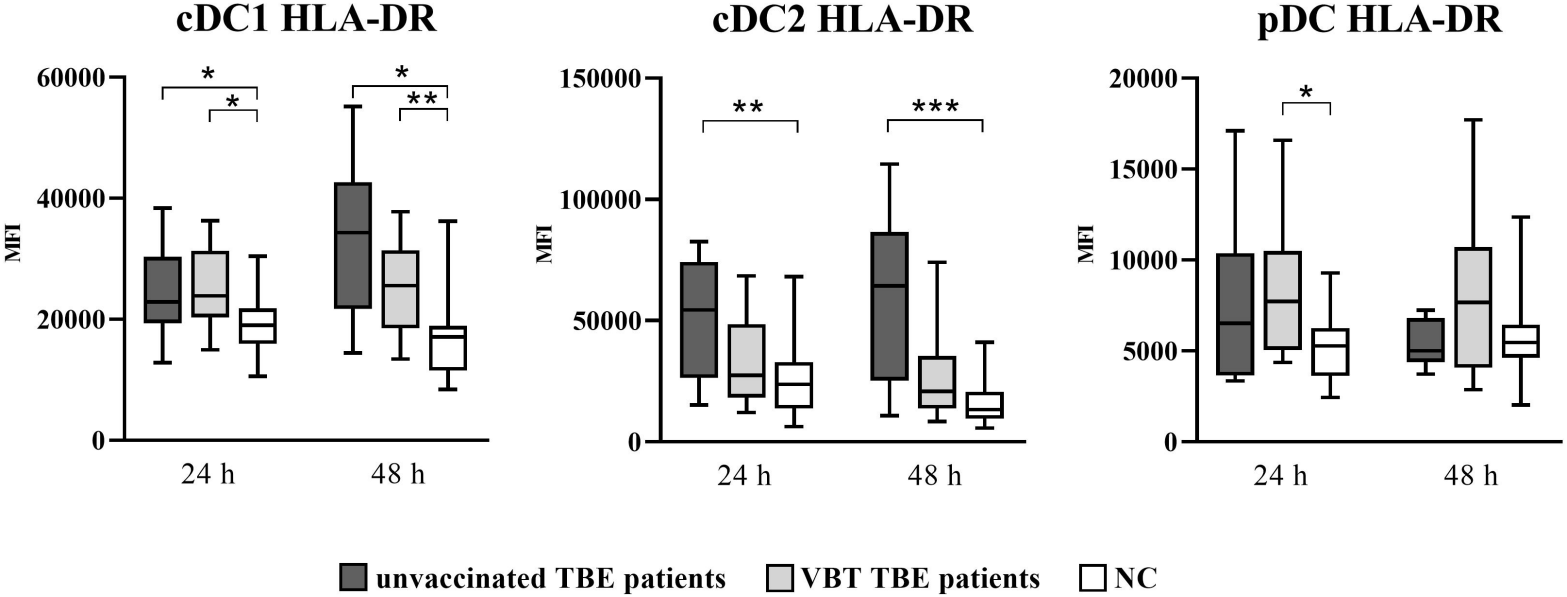
Expression of the activation marker HLA-DR on cDC1, cDC2, and pDC after stimulation with TBEV. To analyze the activation status of DC subpopulations upon *in vitro* TBEV stimulation, PBMCs were stimulated with TBEV and MOI 2, stained with mAb binding the activation/maturation marker HLA-DR for 24 and 48 hours, and analyzed with flow cytometry. The graphs show the MFI value of the respective marker. The box and whisker plots show the median (horizontal line), quartiles (box) and the whiskers, which extend to the most extreme data point. Only statistically significant differences are shown. *p ≤ 0.05, **p ≤ 0.01, and ***p ≤ 0.001. NC, negative control; MFI, median fluorescence intensity; h, hour.

As shown in **Figure 1**, cDC1 from both VBT as well as unvaccinated TBE patients had statistically significant higher HLA-DR MFI at 24 and 48 hours after stimulation with TBEV, whereas only cDC2 from unvaccinated patients had significantly higher HLA-DR expression compared to unstimulated cells. On the other hand, despite the observed trend of higher HLA-DR MFI on stimulated pDC from both groups of patients, the difference compared to unstimulated cells was statistically significant only in VBT TBE patients.

**Figure 2:**
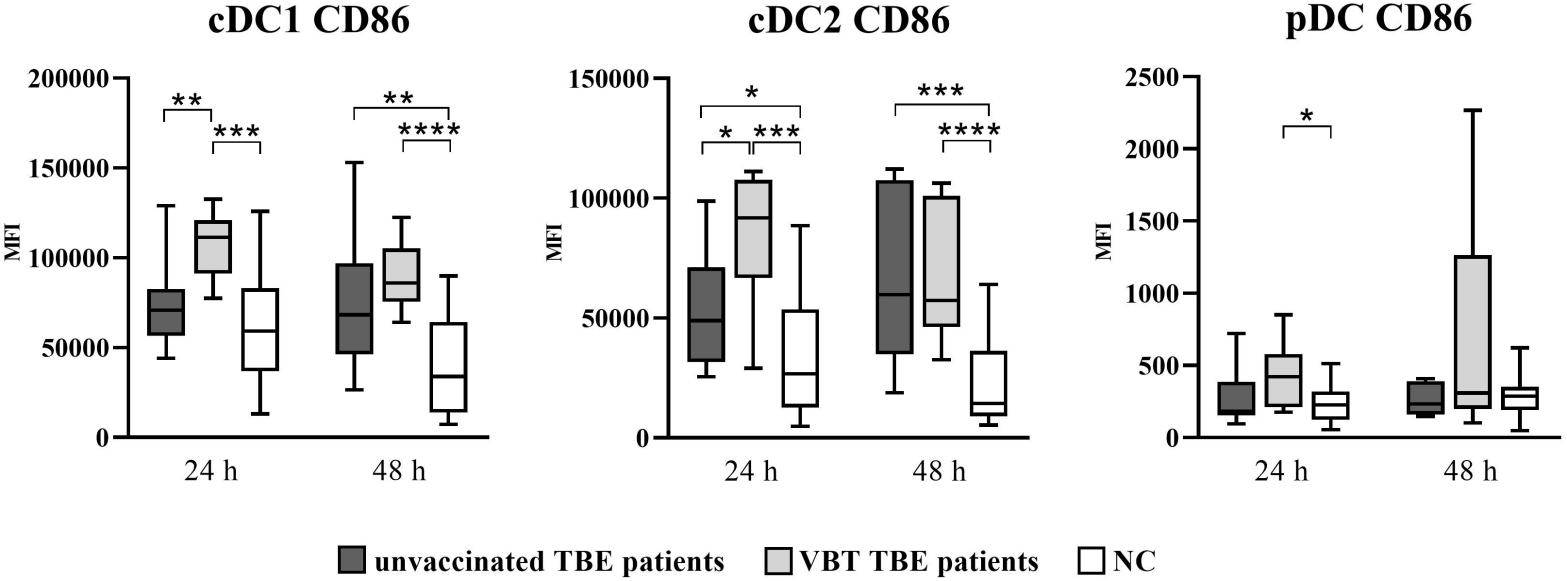
Expression of the activation marker CD86 on cDC1, cDC2, and pDC after stimulation with TBEV. To analyze the activation status of DC subpopulations after *in vitro* TBEV stimulation, PBMCs were stimulated with TBEV MOI 2 for 24 and 48 hours, stained for the activation/maturation marker CD86, and analyzed with flow cytometry. The graphs show the MFI value of the respective marker. The box and whisker plots show the median (horizontal line), quartiles (box) and the whiskers, which extend to the most extreme data point. Only statistically significant differences are shown. *p ≤ 0.05, **p ≤ 0.01, ***p ≤ 0.001, and ****p ≤ 0.0001. NC, negative control; MFI, median fluorescence intensity; h, hour.

Similarly, upregulation of CD86 was also observed on DC after 24- and 48-hour *in vitro* stimulation with TBEV, with significantly higher CD86 MFI detected on cDC1 and cDC2 of both patient groups (**Figure 2**). In addition, the expression of CD86 was significantly higher at 24 hours on cDC2 of VBT patients compared to nonvaccinated TBE patients. On the other hand, a significantly higher CD86 MFI on pDC compared to unstimulated cells was observed only in VBT patients.

Thus, TBEV stimulation of PBMC *in vitro* resulted in significant DC activation observed by profound upregulation of surface activation molecules, especially on cDC1 and cDC2 from both groups of TBE patients, indicating possible involvement of DC in the early immune response during TBEV stimulation.

### 3.2 Differential cytokine expression *in vitro* after TBEV stimulation

To investigate the innate immune response to TBEV at early stages of pathogenesis, we analyzed the production of the pro-inflammatory cytokines IFN-α, IL-1β, IL-6, IL-12 (p70), IL-15, and TNF-α after *in vitro* stimulation of PBMCs. PBMCs were stimulated with TBEV (MOI 2) for 24 hours, 48 hours, and 6 days. At each time point, supernatants were collected and cytokines were analyzed with a multiplex immunoassay on a Magpix instrument. Supernatants from untreated cells served as negative controls.

**Figure 3:**
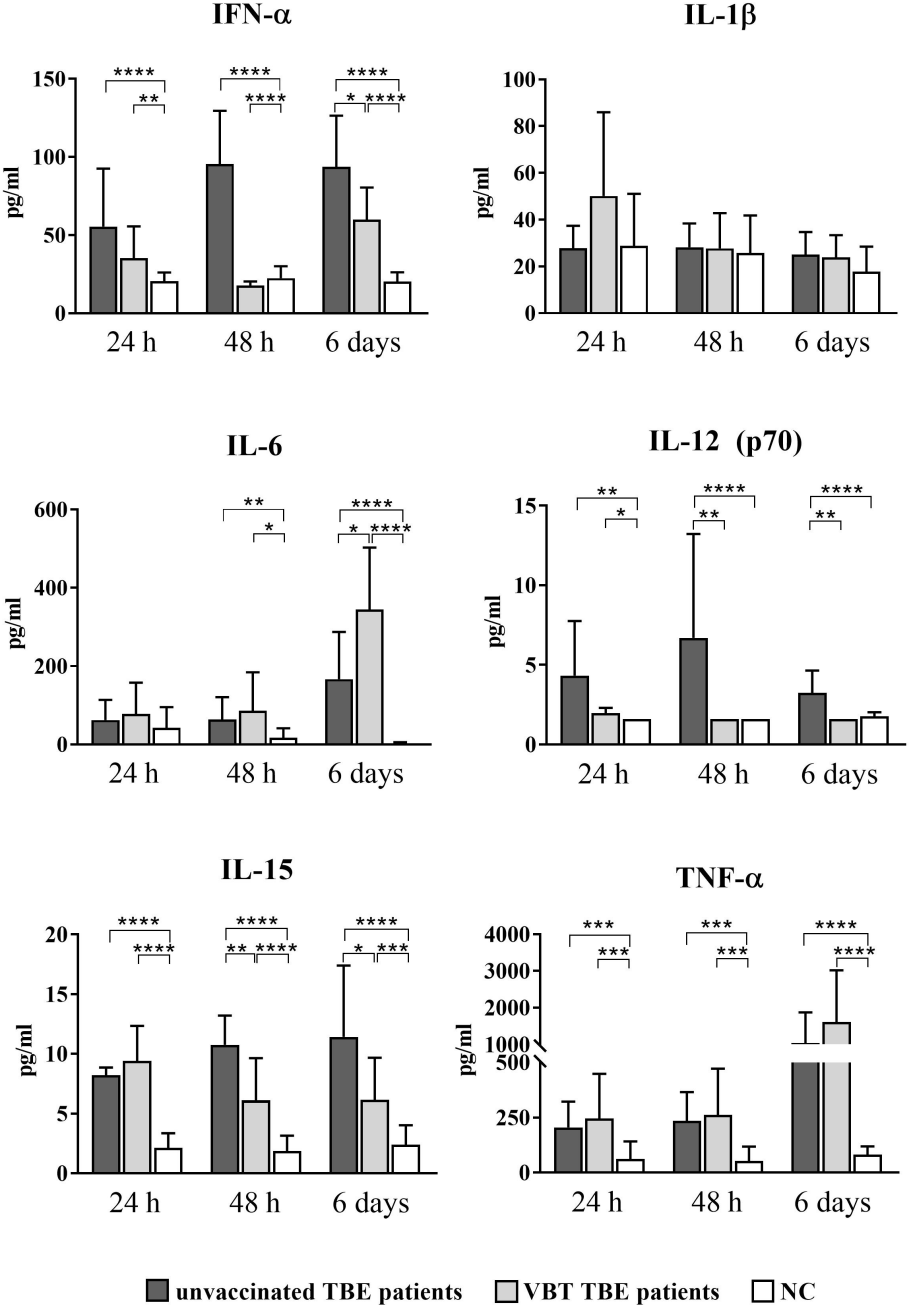
Cytokine expression after stimulation of PBMCs from VBT and unvaccinated TBE patients with TBEV. Supernatants of PBMC were harvested 24 hours, 48 hours, and 6 days post stimulation, and induction of IFN-α, IL-1β, IL6, IL-12 (p70), IL-15, and TNF-α was analyzed with a multiplex immunoassay. Untreated cells served as negative control (NC). The graphs show the concentration of each cytokine in pg/ml. The column plots show the mean with SD. Only statistically significant differences are shown. *p ≤ 0.05, **p ≤ 0.01, ***p ≤ 0.001, and ****p ≤ 0.0001.

As early as 24 hours after stimulation with TBEV *in vitro*, statistically significant upregulation of IFN-α, IL-12 (p70), IL-15, and TNF-α was observed in the supernatants of stimulated PBMCs from VBT and unvaccinated patients compared to unstimulated cells (**Figure 3**). In addition, IL-15 and TNF-α remained significantly elevated after 48-hour PBMC stimulation with TBEV compared to unstimulated cells from both groups of patients. Secretion of IFN-α and IL-12 (p70) was not significantly increased in stimulated PBMCs from VBT patients, whereas it was significantly higher in stimulated PBMCs from unvaccinated patients compared to unstimulated cells. Moreover, the concentrations of IFN-α, IL-12 (p70), and IL-15 measured in PBMC cultures from VBT patients at 48 hours and 6 days were significantly lower compared to unvaccinated patients. In addition, IL-6 was upregulated in the supernatants of stimulated PBMCs from both patient groups at all time points and was significantly higher compared to unstimulated cells 48 hours and 6 days post stimulation. However, PBMCs from VBT patients produced significantly higher levels of IL-6 than in unvaccinated patients after 6 days of *in vitro* stimulation. A trend toward increased TNF-α was also observed in the supernatants of stimulated PBMC from VBT patients at all time points, especially 6 days after stimulation, whereas the concentration of IL-1β did not increase in PBMC cultures from both groups of TBE patients.

Lower IFN-α, IL-12 (p70), and IL-15 levels measured 24 hours and 48 hours after *in vitro* stimulation of PBMCs indicate a weaker innate immune response early after TBEV contact, whereas higher expression of IL-6 and TNF-α at 6 days suggests a more extensive inflammatory response in the late stages of TBEV encounter in VBT patients compared to unvaccinated TBE patients.

## 4 Discussion

TBE is a disease of the CNS caused by TBEV. Because no etiologic treatment for TBE is available, the most effective protection against TBE is regular vaccination. Although the vaccine is highly immunogenic, vaccine breakthrough TBE cases have been reported in several European countries, including Slovenia (10). Despite increasing evidence of immune system involvement in immunopathological manifestations of TBE, a sufficient immune response in the early stages of infection importantly suppresses viral replication and spread (14, 15). Some of the first cells encountering TBEV during natural infection are dendritic cells, which represent an important link between innate and adaptive immunity. To gain insight in the pathogenesis of TBE, we stimulated PBMCs from vaccinated and unvaccinated TBE patients *in vitro* with TBEV and measured DC cell activation by expression of activation and maturation markers and cytokine production.

We found that *in vitro* TBEV stimulation of PBMCs from vaccinated and unvaccinated TBE patients increased the expression of the activation molecules HLA-DR and CD86 on DCs, particularly on a subpopulation of cDCs. cDC cells are the main antigen-presenting cells capable of recognizing both viral single- and double-stranded RNA *via* Toll-like receptors (TLR) 3 and 8, resulting in differential cytokine secretion leading to T cell proliferation and activation. In addition to producing IFN I, cDC1 cells are also known to produce large amounts of IFN III, whereas cDC2 cells produce higher amounts of the inflammatory cytokines IL-6, IL-8, and TNF-α (17). In both groups of TBE patients, cDC1 cells displayed a mature phenotype by significantly increasing the expression of HLA-DR and CD86 compared to unstimulated cells. On the other hand, we observed a significantly higher CD86 MFI on cDC2 from vaccinated TBE patients compared to unvaccinated patients 24 hours post stimulation, indicating a more extensive costimulatory function of this subpopulation of cells in vaccinated patients during the early phase of the anti-viral immune response. However, in contrast to unvaccinated TBE patients, we did not observe a statistically significant difference in HLA-DR MFI on stimulated cDC2 from vaccinated compared to unstimulated PBMCs. Our findings indicate that although CD86 expression on cDC2 is significantly increased in VBT TBE patients, this cell subset’s antigen presentation function may be impaired, potentially hindering the priming of T cells and promoting viral pathogenesis. Studies have shown that several flaviviruses, including JEV (20), DENV (21), WNV (22), and LGTV (23), have been found to impair DC maturation, with immature DCs showing reduced capacity to induce T cell proliferation. As HLA-DR upregulation is observed in unvaccinated patients, we hypothesize that reduced HLA-DR expression on cDC2s in vaccinated patients may be a characteristic of this group, rather than a consequence of viral manipulation of DC function. However, to fully comprehend the capabilities of cDC2s in inducing T cell priming and proliferation among vaccinated TBE patients, more studies are necessary. Similarly, it remains to be investigated whether cDC1 cells from VBT patients may be able to compensate for the diminished antigen presentation by cDC2 cells and play a dominant role in this regard.

Additionally, VBT patients responded with significantly lower concentrations of IFN-α, IL-12p70, and IL-15 compared to unvaccinated patients 48 hours after the initiating stimulation. IFN-α plays a central role in inhibiting viral replication through interferon-stimulated genes (ISG) with antiviral activity (24, 25), as well as by activating other immune cells and inducing secretion of proinflammatory cytokines (26). The absence of an IFN I response in mice infected with Langat virus (LGTV) resulted in uncontrolled viral replication in all organs, early disruption of BBB, and a fatal outcome (27). Similar to other flaviviruses, TBEV has developed various evasion strategies to circumvent the host immune system. One of them is the active reduction of IFN I production, which, however, is limited to the first 24 hours of infection (28, 29). Therefore, we hypothesize that a reduced early IFN-α response is a characteristic of VBT patients that could lead to higher viral load and viral dissemination, contributing to earlier neuroinvasion and onset of disease symptoms.

IFN-α, in combination with IL-6 and BAFF, also promotes differentiation of B cells into plasma cells and antibody production after stimulation with inactivated TBEV *in vitro* (30). The amount of IFN-α production has been shown to influence the antibody response to yellow fever vaccine (31) and influenza vaccine (32). Older individuals develop a lower antibody response after vaccination against TBE (33), and antibody levels induced by TBE vaccination decline more rapidly over time compared to younger individuals (34–37). Patients with VBT TBE are usually older and often develop illness before the recommended time for booster vaccination (10). Therefore, the lower IFN-α response observed upon stimulation of PBMCs obtained from patients with VBT TBE could potentially be a contributing factor to an inadequate antibody response following TBE vaccination and shorter protection against the disease than expected. Nonetheless, vaccinated TBE patients develop a more robust antibody response after infection with TBEV in comparison to unvaccinated patients (10–12), indicating that vaccination induced the formation of memory B cells that differentiate into antibody-producing plasma cells after exposure to TBEV. However, the immune response in VBT patients was presumably not fast or effective enough to prevent disease.

Upon activation, DCs also upregulate the production of several proinflammatory cytokines, including IL-6, IL-12, IL-15, and TNF-α (17). In this study, PBMCs obtained from patients with VBT TBE responded with lower IL-12 (p70) and IL-15 production after 2 and 6 days of stimulation with TBEV compared to unvaccinated patients, suggesting a weaker functional capacity of DCs in VBT patients in the early stages of TBEV stimulation. Both IL-12 and IL-15 are known activators of NK cells, which control infection through the secretion of perforin and granzyme B and production of IFN-γ and TNF-α (38). Blom et al. (39) have shown that TBEV likely triggers NK cell activation at an earlier stage of infection because elevated Ki67 levels in NK cells were associated with elevated levels of IFN-γ and TNF-α in plasma from TBE patients. In addition, NK cells are thought to significantly influence the course and outcome of the disease caused by other flaviviruses, including Dengue virus (40, 41), West Nile virus (42), and Japanese encephalitis virus (43), as well as vaccination with live attenuated yellow fever virus (44). Therefore, lower expression of IL-12 (p70) and IL-15 in VBT patients could lead to lower NK cell activation, promoting viral dissemination at an early stage of infection. However, to better understand the involvement of NK cells in VBT TBE, further studies investigating the activation and effector function of this immune cell subset are needed.

*In vitro* stimulation of PBMCs with TBEV showed that, in addition to lower proinflammatory response, there was a marked upregulation of the proinflammatory cytokines IL-6 and TNF-α in VBT patients compared to unvaccinated patients after 6 days of PBMC stimulation *in vitro*, which was statistically significant in the case of IL-6. Although the production of IL-6 is generally associated with a protective immune response, it could also contribute to an increased inflammatory response and disease progression. Elevated levels of IL-6 have been measured in clinical samples from patients that developed a more severe form of Japanese encephalitis (45). Excessive synthesis of IL-6 in combination with other inflammatory mediators such as TNF-α is thought to lead to increased BBB permeability and neuronal damage (46–49). The more extensive disruption of the BBB could facilitate the entry of virus and immune cells to the CNS and consequently contribute to a more severe manifestation of the disease in VBT patients.

Our *in vitro* results show that VBT and unvaccinated TBE patients upregulate the expression of activating molecules on DCs, particularly cDCs, to a similar extent, but differ in cytokine production after stimulation with TBEV. PBMCs from VBT patients responded with lower levels of IFN-α and the proinflammatory cytokines IL-12 (p70) and IL-15 after *in vitro* stimulation, which could facilitate viral replication and lead to poorer activation of other immune cells. On the other hand, the measured elevated levels of IL-6 as well as observed trend of higher expression of TNF-α after 6 days of PBMC stimulation could possibly contribute to disruption of the BBB and facilitate the influx of TBEV and immune cells into the CNS, which in turn could contribute to a more severe disease.

Although this study has provided valuable insight into the immune response of VBT TBE patients, certain limitations remain. Specifically, the lack of samples from VBT patients prior to their encounter with TBE virus and subsequent development of TBE impedes our ability to fully understand the immune status of these individuals at the time of tick bite. There is a limited number of samples available from VBT patients in this study, which may limit the generalizability of the findings. Additionally, the inclusion of a control group consisting of vaccinated individuals who have been exposed to the virus but remain asymptomatic would provide crucial information regarding the immune system’s ability to combat TBEV. However, practical constraints make it difficult to obtain blood samples from such individuals, as they are unlikely to seek medical attention. Moreover, it should be emphasized that investigating other cell types, in addition to dendritic cells, may be crucial to gain a complete understanding of the immune response to TBEV and the mechanisms involved in VBT TBE.

Despite these limitations, this study is the first and only VBT TBE study to date, and the samples collected are extremely rare and valuable. The findings of this study provide important insights into the immune response of individuals who develop VBT TBE, which can inform future research and potentially lead to the development of more effective treatments and prevention strategies of TBE.

## Data availability statement

The original contributions presented in the study are included in the article/**Supplementary Material**. Further inquiries can be directed to the corresponding author.

## Ethics statement

The studies involving human participants were reviewed and approved by National Medical Ethics Committee (approval no. 0120-564/2018/13). The patients/participants provided their written informed consent to participate in this study.

## Author contributions

MM, MK, NK and TAZ designed the study. PB and FS included patients in the study and obtained clinical information. MM performed the experiments and formal analysis. MM, MK, NK, TAZ, ANK, AI, PB, and FS performed review and editing. MM performed the data analysis and interpretation. MM wrote the original draft. MM, MK, NK, TAZ, ANK, AI, PB and FS performed review and editing. All authors contributed to the article and approved the submitted version.
