# Supplementary material for: Dendritic cell activation and cytokine response in vaccine breakthrough TBE patients after in vitro stimulation with TBEV
Source: Front Immunol. 2023 May 16;14:1190803. doi: 10.3389/fimmu.2023.1190803 (PMC10228714; doi:10.3389/fimmu.2023.1190803)
Supplement: Supplementary file 1 [file DataSheet_1.docx]

Supplementary Material

Dendritic cell activation and cytokine response in vaccine breakthrough TBE patients after in vitro stimulation with TBEV

**Miša Marušić, Andreja Nataša Kopitar, Miša Korva, Nataša Knap, Petra Bogovič, Franc Strle, Alojz Ihan, Tatjana Avšič-Županc***

*** Correspondence:** Tatjana Avšič Županc: tatjana.avsic@mf.uni-lj.si


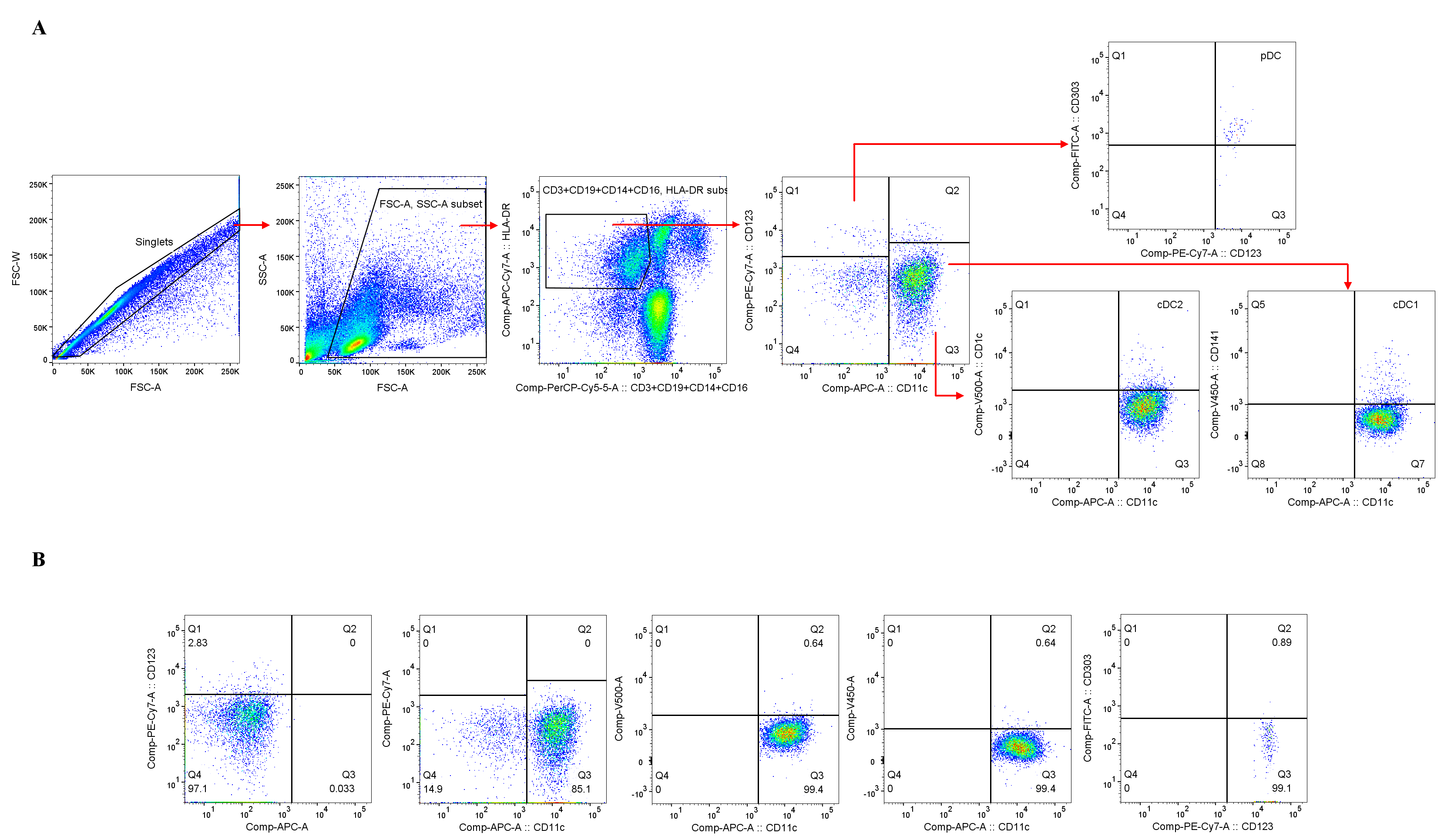


**Supplementary Figure 1. A-**Gating strategy for multicolor flow cytometric detection of total dendritic cells (DCs) and their subsets after *in vitro* PBMC stimulation with TBEV. At first, doublets and aggregates were avoided by selecting singlet cells on a forward scatter: FSC (area)/FSC (height) plot. Next, leukocytes were gated by forward scatter characteristic (FSC) versus side scatter characteristics (SSC). Cells that were HLA-DR^+^ and lineage^-^ (CD3 CD19 CD14 CD16) were further divided into conventional DCs (CD11+) subtypes cDC1 (CD11c+CD141+), cDC2 (CD11cCD1c+) and plasmacytoid dendritic cells pDC (CD123+CD303). The level ofactivation of all three DC subsets was assessed by measuring the Mean Fluorescent Intensity (MFI) of CD86 and HLA-DR, respectively. **B**-Fluorescence-minus-one (FMO) controls for DC activation after *in vitro* PBMC stimulation with TBEV. Fluorescence minus one (FMO) controls were prepared without adding a particular fluorochrome-conjugated antibody.
